# Continued versus Interrupted Targeted Therapy during Metastasis-Directed Stereotactic Radiotherapy: A Retrospective Multi-Center Safety and Efficacy Analysis

**DOI:** 10.3390/cancers13194780

**Published:** 2021-09-24

**Authors:** Stephanie G. C. Kroeze, Corinna Fritz, Jana Schaule, Oliver Blanck, Klaus Henning Kahl, David Kaul, Shankar Siva, Sabine Gerum, An Claes, Nora Sundahl, Sonja Adebahr, Susanne Stera, Markus M. Schymalla, Nasrin Abbasi-Senger, Daniel Buergy, Michael Geier, Marcella Szuecs, Fabian Lohaus, Guido Henke, Stephanie E. Combs, Matthias Guckenberger

**Affiliations:** 1 Radiation Oncology, University Hospital Zürich, 8011 Zurich, Switzerland; fricor@posteo.de (C.F.); jana.schaule@usz.ch (J.S.); matthias.guckenberger@usz.ch (M.G.); 2 Radiation Oncology, University Medical Center Schleswig-Holstein, 24105 Kiel, Germany; blanck@saphir-rc.com; 3 Radiation Oncology, University Hospital Augsburg, 86156 Augsburg, Germany; KlausHenning.Kahl@uk-augsburg.de; 4 Radiation Oncology, Charité-University Hospital Berlin, 12200 Berlin, Germany; david.kaul@charite.de; 5 Radiation Oncology, Peter MacCallum Cancer Centre, University of Melbourne, Melbourne 3010, Australia; shankar.siva@petermac.org; 6 Radiation Oncology, University Hospital Munich, 80336 Munich, Germany; s.gerum@salk.at; 7 Radiation Oncology, University Medical Center Utrecht, 3584 CX Utrecht, The Netherlands; a.claes@umcutrecht.nl; 8 Radiation Oncology, University Hospital Ghent, 9000 Ghent, Belgium; nora.sundahl@ugent.be; 9 Department of Radiation Oncology, University Medical Center Freiburg, Faculty of Medicine, University of Freiburg, 79106 Freiburg, Germany; sonja.adebahr@uniklinik-freiburg.de; 10 German Cancer Consortium (DKTK) Partner Site Freiburg, German Cancer Research Center (DKFZ), 69120 Heidelberg, Germany; 11 Radiation Oncology, University Hospital Frankfurt, 60318 Frankfurt, Germany; susanne.stera@kgu.de; 12 Radiation Oncology, Philipps-University Marburg, 35043 Marburg, Germany; markusmichael.schymalla@uk-gm.de; 13 Radiation Oncology, University Hospital Jena, 07743 Jena, Germany; Nasrin.Abbasi-Senger@helios-gesundheit.de; 14 Radiation Oncology, Universitätsmedizin Mannheim, Medical Faculty Mannheim, Heidelberg University, 68167 Mannheim, Germany; daniel.buergy@medma.uni-heidelberg.de; 15 Radiation Oncology, Ordensklinikum Linz, 4020 Linz, Austria; michael.geier@radio-log.de; 16 Radiation Oncology, University Hospital Rostock, 18059 Rostock, Germany; marcella.szuecs@uni-rostock.de; 17 Radiation Oncology, University Hospital Tübingen, 72076 Tübingen, Germany; Fabian.Lohaus@uniklinikum-dresden.de; 18 Radiation Oncology, Kantonsspital St. Gallen, 9007 St. Gallen, Switzerland; guido.henke@kssg.ch; 19 Radiation Oncology, Technical University Munich, 81675 Munich, Germany; stephanie.combs@tum.de

**Keywords:** stereotactic, metastasis-directed radiotherapy, targeted therapy, concurrent, tyrosine kinase inhibitors, BRAF inhibitors

## Abstract

**Simple Summary:**

The increasing use of targeted therapy (TT) has resulted in prolonged disease control and survival in many metastatic cancers. In parallel, stereotactic radiotherapy (SRT) is increasingly performed in patients receiving TT to obtain a durable control of resistant metastases, and thereby to prolong the time to disseminated disease progression and switch of systemic therapy. The aim of this study was to analyze the safety and efficacy of SRT combined with TT in metastatic cancer patients and to assess the influence of continuous vs. interrupted TT during metastasis-directed SRT. The data of 454 SRTs in 158 patients from the international multicenter database (TOaSTT) on metastatic cancer patients treated with SRT and concurrent TT (within 30 days) were analyzed. We found that there was no significant difference in survival, progression, or severe toxicity, whether TT was interrupted during SRT or not. Although any-grade acute and late SRT-related toxicity occurred in 63 (40%) and 52 (33%) patients, severe SRT-related toxicity rates were low (3% and 4%, respectively). The highest toxicity rates were observed for the combination of SRT and EGFRi or BRAF/MEKi, and any grade of toxicity was significantly increased when EGFRi or BRAF/MEKi were continued during SRT. However, this did not account for severe toxicity.

**Abstract:**

The increasing use of targeted therapy (TT) has resulted in prolonged disease control and survival in many metastatic cancers. In parallel, stereotactic radiotherapy (SRT) is increasingly performed in patients receiving TT to obtain a durable control of resistant metastases, and thereby to prolong the time to disseminated disease progression and switch of systemic therapy. The aims of this study were to analyze the safety and efficacy of SRT combined with TT in metastatic cancer patients and to assess the influence of continuous vs. interrupted TT during metastasis-directed SRT. The data of 454 SRTs in 158 patients from the international multicenter database (TOaSTT) on metastatic cancer patients treated with SRT and concurrent TT (within 30 days) were analyzed using Kaplan–Meier and log rank testing. Toxicity was defined by the CTCAE v4.03 criteria. The median FU was 19.9 mo (range 1–102 mo); 1y OS, PFS and LC were 59%, 24% and 84%, respectively. Median TTS was 25.5 mo (95% CI 11–40). TT was started before SRT in 77% of patients. TT was interrupted during SRT in 44% of patients, with a median interruption of 7 (range 1–42) days. There was no significant difference in OS or PFS whether TT was temporarily interrupted during SRT or not. Any-grade acute and late SRT-related toxicity occurred in 63 (40%) and 52 (33%) patients, respectively. The highest toxicity rates were observed for the combination of SRT and EGFRi or BRAF/MEKi, and any-grade toxicity was significantly increased when EGFRi (*p* = 0.016) or BRAF/MEKi (*p* = 0.009) were continued during SRT. Severe (≥grade 3) acute and late SRT-related toxicity were observed in 5 (3%) and 7 (4%) patients, respectively, most frequently in patients treated with EGFRi or BRAF/MEKi and in the intracranial cohort. There was no significant difference in severe toxicity whether TT was interrupted before and after SRT or not. In conclusion, SRT and continuous vs. interrupted TT in metastatic cancer patients did not influence OS or PFS. Overall, severe toxicity of combined treatment was rare; a potentially increased toxicity after SRT and continuous treatment with EGFR inhibitors or BRAF(±MEK) inhibitors requires further evaluation.

## 1. Introduction

Targeted therapies (TT) are increasingly used in metastatic cancer patients, and they have become the standard first line of therapy for several tumor types. This has resulted in a prolonged disease control and patient survival rate compared to chemotherapy [1,2,3]. However, complete and durable responses are rarely observed, and most patients will inherently develop acquired drug resistance, followed by disease progression [4,5]. Disease progression under treatment with TT is most frequently located in the initially involved sites [6,7], paving the way for a multidisciplinary approach with the inclusion of a metastasis-directed therapy, which is currently increasingly performed. 

With our expanding knowledge on the diversity of metastatic disease, metastasis-directed stereotactic radiotherapy (SRT) has shown to achieve durable local metastases control and to possibly prolong the time to systemic disease progression and time to switch of systemic therapy to the next line of treatment [8,9]. However, due to the rapid introduction of novel targeted drugs into routine patient care, there is still limited knowledge on the safety and efficacy of combined SRT and TT, and whether interruption of TT during SRT delivery influences the safety and efficacy profile [10]. An international survey reported that TT was most frequently interrupted for a median of one week prior to and after SRT [11]. However, a rapid development of tumor flare after TT interruption has been observed in the literature [12]. The aim of this retrospective multicenter registry study was to examine the safety and efficacy of interrupted vs. continued TT during SRT in patients with metastatic cancer.

## 2. Materials and Methods

This analysis is part of an international multicenter registry study (TOaSTT database), which collected stage IV cancer patients treated with SRT and concurrent TT or immunotherapy. The project was initiated in the working group radiosurgery and stereotactic radiotherapy of the German Society for Radiation Oncology (DEGRO), and approval of the study was obtained from ethics committees at all participating sites (BASEC-Nr. 2016-01807). 

All adult (≥18 years old) patients with metastatic disease treated with SRT concurrently to TT were eligible for study inclusion. Targeted therapy is defined as drugs or substances that target specific molecules involved in tumor growth and metastasis formation [13]. Concurrent treatment was defined as treatment with TT within 30 days before or after SRT. SRT of brain metastases was defined as delivery of a maximum of 5 fractions and a minimum total dose of 16 Gy. Stereotactic body radiotherapy (SBRT) was defined as the delivery of ≤10 fractions with a minimum total dose of 50 Gy (2 Gy equivalent, α/β of 10 Gy). Radiotherapy dose, the decision whether to interrupt TT, as well as the length of interruption of TT during SRT, which was defined as any change of drug application with an interval of systemic therapy free time around SRT delivery, was at the discretion of the participating center.

The primary endpoint of this study was safety of combined modality treatment. Secondary endpoints were one year and overall survival (OS), progression free survival (PFS), local metastases control (LC), and freedom from systemic therapy switch (TTS). Acute (<3 months following SRT) and late (≥3 months after SRT) severe toxicity (grade ≥ 3 events) were evaluated using the Common Terminology Criteria for Adverse Events (CTCAE) v4.03 and were probably or likely attributable to the SRT, or a combination of SRT and TT. OS was defined as the time from SRT to death or last follow-up. PFS and LC were defined as time from SRT to overall or local disease progression and were determined by PET-CT/MRI, MRI, CT-scan, ultrasound, or X-ray imaging at the discretion of the participating center. PFS and LC were evaluated by censoring patients at their most recent imaging. TTS was defined as the time from SRT until the start of a new systemic therapy. Descriptive statistical analysis was performed with SPSS v26.0 statistic software package (IBM Corp., Armonk, NY, USA), using Kaplan–Meier survival curves with log-rank analysis to evaluate survival. The Mann–Whitney test and chi-square test were used to compare differences between groups. A *p*-value of less than 0.05 was regarded statistically significant.

## 3. Results

### 3.1. Patient Characteristics

This analysis was based on 158 patients from 18 participating centers fulfilling the inclusion and exclusion criteria of this study; patients were treated with a total of 454 SRT courses between July 2009 and March 2018 (Table 1). Baseline characteristics were balanced between patients continuing vs. interrupting TT, except for age. Most patients had already received prior local or systemic therapies for their cancer (98%) and had metastatic disease with an involvement of >1 organ (75%). The ECOG performance score was ≤1 in 98% of patients at the time of SRT. Fifty-five percent had oligometastatic disease (defined as ≤5 lesions), and 45% had >5 metastases at the time of SRT.

### 3.2. Targeted Therapy

**Figure 1:**
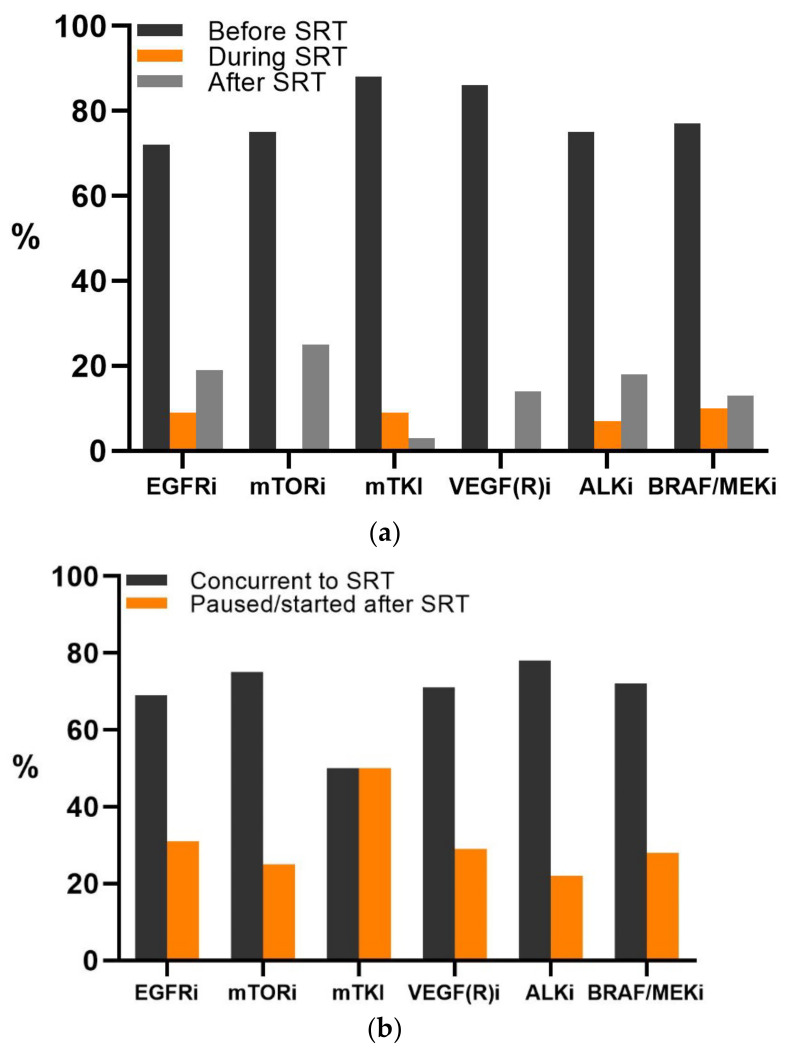
(**a**) Timing of the start of targeted therapy (TT) in relation to stereotactic radiotherapy (SRT) in included patients. (**b**) The number of patients in which TT was interrupted or continued during SRT, as per TT group.

Patients were treated with EGFR inhibitors (EGFRi, 37%), BRAF (±MEK) inhibitors (BRAF/MEKi, 27%), multikinase inhibitors (mTKI, 18.4%), ALK inhibitors (ALKi, 10%), VEGF(R) inhibitors (VEGF(R)i, 4.4%), or mTOR inhibitors (mTORi, 2.5%) (Table S2). The TT had been started before SRT in the majority of patients (77%) (Figure 1a). For these patients, the TT was started at, on average, 195 (range 5–1490) days before SRT, and 68% of the patients continued their TT uninterrupted during SRT (Figure 1b). Interruption of TT was performed most frequently in patients treated with mTKI (50%), and the median TT interruption was a total of 7 days for all patients and forms of TT (range 1–42 days). For patients starting TT after SRT, the median time interval was 7 (range 0–49) days after SRT.

### 3.3. Stereotactic Radiotherapy

The majority of patients were treated with SRT for brain metastases, with a total of 387 metastases treated in 147 patients. A median number of 2 (range 1–11) brain metastases were irradiated per patient. The median GTV volume of brain metastases was 1.0 mL (range 0.03–23.9 mL). The median SRT dose (BED10) prescribed to the planning targeted volume (PTV) was 63 Gy (range 44–114 Gy) in median 1 fraction (range 1–6 fractions). Sixty-seven extracranial metastases were treated with SBRT in 56 patients, with a median of 1 (range 1–3) metastasis per treatment session. SRT-treated lesions were located in the bone (*n* = 34), lung (*n* = 27), liver (*n* = 10), soft tissue (*n* = 4), adrenal gland (*n* = 3), or lymph nodes (*n* = 2). The median GTV volume was 7.5 mL (range 0.54–154.5 mL), and the median prescribed SRT dose (BED10) was 93 Gy (range 53–180 Gy). The median number of fractions was 3 (range 1–8) per SBRT session.

### 3.4. Efficacy

The median follow-up was 19.9 months (range 1–102 months). One-year survival was 59%. The cause of death was cancer-related in 88.3% of patients. One-year LC and PFS were 84% and 24%, respectively.

**Figure 2:**
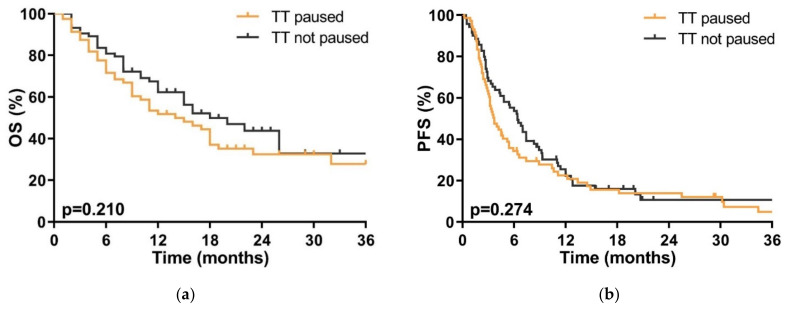
(**a**) Overall survival (OS) of patients where targeted therapy (TT) was interrupted (orange line) or continued (gray line) during stereotactic radiotherapy (SRT). *p* < 0.05 is statistically significant. (**b**) Progression free survival (PFS) in patients where targeted therapy (TT) was interrupted (orange line) or continued (gray line) during stereotactic radiotherapy (SRT). *p* < 0.05 is statistically significant.

There was no significant difference in OS whether TT was continued during SRT (median 18 months (95% CI 11–25)) or whether it was interrupted (14 months (95% CI 7–20), *p* = 0.210) during SRT; there was also no significant difference in PFS with 6.4 months (95% CI 4.9–7.9) vs. 3.6 months (95% CI 2.6–4.6, *p* = 0.274) (Figure 2).

After 1 year, 64% of patients still received the same TT as at the time of SRT, with a median time of 25.5 months until TTS (95% CI 11–40 months). In patients with progressive disease, the next line of treatment was repeat radiotherapy in 56%, with another SRT in 35% of cases, and conventionally fractionated radiotherapy in 21% of cases. Organs treated with conventionally fractionated radiotherapy or SRT in case of progression were brain (15%), lymph nodes (11%), lung (25%), abdomen (15%), bone (29%), and soft tissue (6%). Fifty percent of these patients showed symptomatic progressive disease. Thirty-six percent of patients switched to a new TT or immune checkpoint inhibition, 28% switched to chemotherapy, and 7% were treated with surgery. 

### 3.5. Toxicity

Any-grade acute and late toxicity caused or worsened by SRT was observed in 63 (40%) and 52 (33%) patients, respectively. Acute severe (≥G3) toxicity was observed in 1.6% of patients and late severe toxicity in 4.9%. Severe toxicity was primarily observed after SRT of brain metastases, consisting mainly of G3 neurocognitive problems (*n* = 6) as well as G3 cerebral necrosis (*n* = 3). Only two G3 toxicities following SRT were observed (Table S1), consisting of two patients with dyspnea after SRT of pulmonary metastasis during treatment with EGFRi. One G5 late toxicity, probably caused by SRT, was a thromboembolic event after SBRT of a pulmonary metastasis in a patient receiving EGFRi. In 4.5% of patients that developed acute toxicity there was a change in therapy management, consisting of a TT interruption in 3%, TT dose reduction in 1%, and radiation dose reduction in 0.5% of patients. Overall, there was no significant correlation of the occurrence of toxicity to any of the specific clinical variables (Table 2). 

**Figure 3:**
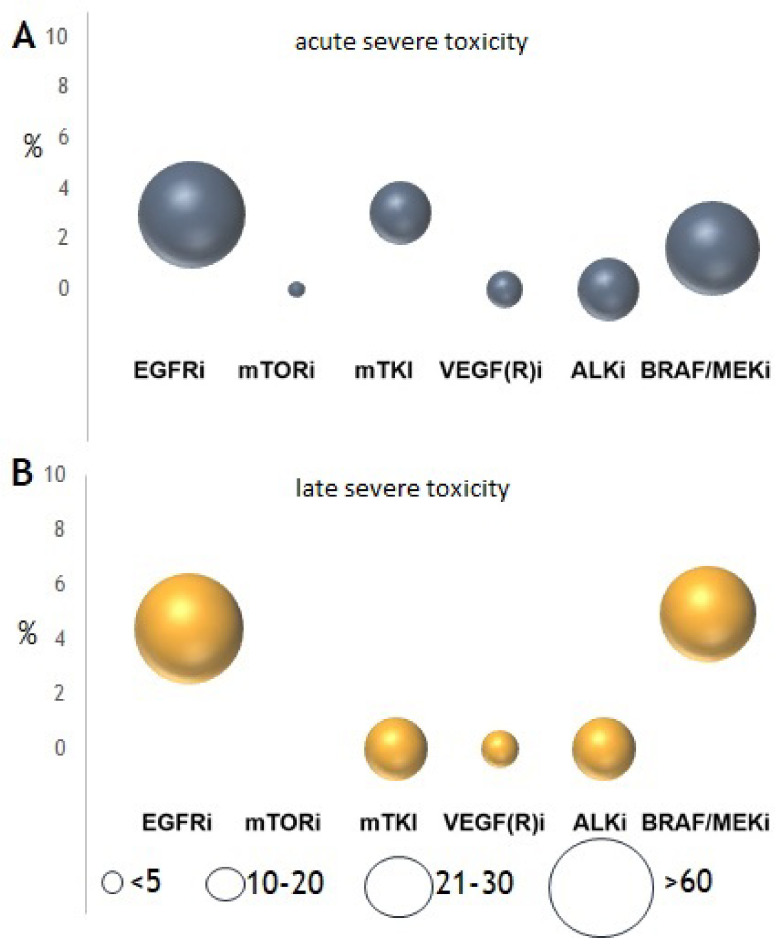
Observed (**A**) acute (≤3 months) and (**B**) late (>3 months) severe (CTCAE v4.03 ≥ G3) toxicity after stereotactic radiotherapy (SRT). Size of the circle reflects the number of included patients.

**Figure 4:**
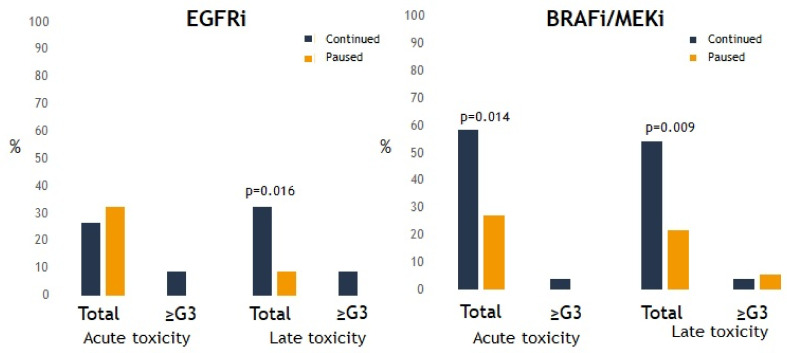
Presence of any-grade or severe toxicity after stereotactic radiotherapy (SRT) for every targeted therapy (TT) group. *p* < 0.05 is statistically significant.

When analyzing the specific types of TT, most acute and late toxicities were observed in patients treated with EGFRi or BRAFi/MEKi (Figure 3). Sixteen percent of any-grade lung toxicity, 4.4% of any-grade bone toxicity, and 31% of any-grade CNS toxicity were observed in patients receiving EGFRi. In patients receiving BRAFi/MEKi, 34% had CNS toxicity and 0% had extracranial toxicity. For these two groups of drugs, the risk of any grade of toxicity was significantly higher when these were continued during SRT. However, there was no significant difference in severe toxicity whether or not EGFRi or BRAFi/MEKi was interrupted during SRT (Figure 4). Furthermore, there was no significant difference in the development of any-grade (*p* = 0.443) or severe toxicity (*p* = 0.167) for patients receiving BRAFi monotherapy (*n* = 15) compared to BRAFi/MEKi (*n* = 28).

## 4. Discussion

In this multicenter real-world retrospective study, we report on the observed toxicity and efficacy of 158 patients treated with SRT for 454 metastatic lesions concurrent to TT. In was observed that the addition of SRT to patients receiving TT resulted in low rates of severe toxicity. Most toxicity was observed when SRT was combined with EGFRi or BRAFi/MEKi. Importantly, there was no significant difference in PFS and OS, as well as severe toxicity, when TT was interrupted or continued during SRT. However, there was a significantly increased risk of any grade of toxicity for patients under EGFRi and BRAFi/MEKi, when continued during SRT. 

The majority of patients will develop resistance to their TT, and chances of survival are reduced for every consecutive line of received TT after resistance occurs [14]. The body of evidence for the efficacy of the addition of SRT in a multimodality concept with TT is slowly growing. Especially for oligometastatic or oligoprogressive disease, SRT appears to be able to delay disease progression and the time to TT switch [15]. Meyer et al. showed, in a large cohort of renal cell carcinoma (RCC) patients, that a median TTS of 13.2 months for patients progressing under TT could be reached [16]. Similar results have been published for non-small cell lung cancer (NSCLC) patients, where a durable local control, improved PFS, and prolonged TTS were observed [15,17,18,19,20]. Our cohort consisted of the primary cancer types that are frequently treated with a combination of SRT and TT: NSCLC, RCC, and melanoma. Here, we even observed a TTS of 25.5 months and, with the majority of patients being progressive under TT, this is another example of this concept appearing to be very effective for selected patients. 

We observed a low level of severe SRT-associated toxicity. This is in accordance with the current literature [10,21]. To possibly prevent toxicity, it remains largely unknown whether it is better to interrupt TT during SRT or not, resulting in a variety of different procedures in centers worldwide. The half-life of TTs ranges from 24 to 57 h, which makes it possible to allow for a quick washout before SRT is performed. However, the occurrence of a rapid tumor-flare after stopping TT for only a couple of days has also been described in the literature [12]. In an international consensus paper on this topic, it appeared that TT was frequently interrupted for approximately one week in most centers [11]. When in our study TT was interrupted, this was also for about one week surrounding the SRT. Interestingly, especially mTKIs were interrupted in 50% of cases, which might be explained by the studies of Staehler et al. and Brade et al. who observed intracerebral hemorrhage and upper GI hemorrhage after concurrent treatment with the mTKIs sorafenib or sunitinib [22,23]. In our study, these side effects of mTKIs were not observed, although the number of patients was relatively small. 

Importantly, we did observe an increased risk of all-grade toxicity when TT was continued during SRT; however, this potential increase in low-grade toxicity did not translate to an increased risk of developing severe toxicity. In summary, our results suggest that the combination of SRT and TT is safe, and that the safety profile is similar whether or not TT is paused during and among SRT delivery.

Of all TTs, SRT-induced side effects were most frequently observed in patients receiving EGFRi or BRAFi/MEKi. For EGFRi, these toxicities occurred both cranially and extracranially in the lungs and bones. Studies that examined the safety of concurrent therapies mainly observed toxicity for extracranial SRT as well, although it should be noted that the available series on cerebral SRT are small [24,25]. EGFRi are mainly applied in EGFR mutant non-small cell lung cancer and colon cancer. Published toxicity data on extracranial SRT consisted mostly of pneumonitis, stomatitis, and esophagitis, similar to our observations [26,27]. EGFR is expressed in epithelial cells where it promotes mucosal repair in the intestines and protects the skin barrier [28]. Within the lungs, EGFR plays an important role in the regeneration of epithelial cells to augment lung fibrosis [29]. When combining SRT to EGFRi in these specific organs, EGFR-mediated repair of the damage to the healthy tissue caused by SRT can therefore be impaired. Besides EGFRi, patients treated with BRAFi/MEKi were characterized by an increased risk of any grade of toxicity when TT was continued during SRT. However, here the risk of severe toxicity was likewise not increased when BRAFi/MEKi was continued. For this combination, mainly an increased risk of skin toxicity has been described in the literature [21]. Although the study of Hecht et al. observed an increased risk in grade ≥2 toxicity and worse OS when BRAFi was continued during SRT due to its radiosensitizing properties, the study of Ziegler et al. did not see these side-effects when BRAFi was combined with MEKi [30,31], with the hypothesis that MEKi can mitigate the occurrence of skin side effects [32]. In this study, we did not observe skin toxicity since the dose to the skin is usually limited with SRT. Furthermore, there was no difference in the occurrence of toxicity when combining SBRT with BRAFi monotherapy compared to BRAFi/MEKi. However, because of the radiosensitization of BRAFi, the combination with a high radiation dose to brain tissue might be more likely to result in an increased risk of cerebral necrosis. 

Limitations of this study lie in its retrospective nature, resulting in non-uniform treatment of SRT and TT. This study used TT as a common factor and purposely included several tumor types to generate more knowledge on the currently limited literature on the topic of combining SRT with TT. However, this is one of the largest datasets on this topic, using valuable real-world current clinical practice, and it can be regarded as a meaningful way of adapting evidence generation in a rapidly changing field with lack of prospective trials. Furthermore, due to the retrospective data collection, some low-grade toxicities were possibly underreported [33]. However, severe toxicity is usually reported, and observed toxicity was comparable to current literature [10,21]. This retrospective database is hypothesis-generating on the still limited literature exploring the efficacy and safety concurrent SRT and TT. Prospective database collection is underway with the prospective collection of toxicity on combined TT and SRT as part of this present study, as well as the Oligocare study, the collaboration project of the EORTC and ESTRO ((NCT03818503, ClinicalTrials.gov).

## 5. Conclusions

In conclusion, high-dose SRT concurrent with targeted therapy was characterized by a favorable safety profile, irrespective of whether TT was interrupted during and among SRT. There was also no difference in OS or PFS whether targeted therapy was interrupted or continued during SRT. The risk of any-grade toxicity was significantly increased when EGFR inhibitors or BRAF(±MEK) inhibitors were continued during SRT compared to when these inhibitors were interrupted at the time of SRT, but the risk of severe toxicity was not.

## Figures and Tables

**Table 1:** Patient characteristics subdivided into patients where targeted therapy (TT) was interrupted during stereotactic radiotherapy and patients where TT was continued.

Patient Characteristics	Total # of Patients (*n* = 158) (%) Median (Range)	Tt Continued *n* = 88 (56%)	Tt Paused *n* = 70 (44%)	*p* Value
Age (y)	60 (26–86)	57 (26–80)	64 (22–86)	0.16
Sex				0.798
Female	58 (37)	28 (35)	27 (37)	
Male	100 (63)	52 (65)	46 (63)	
Primary tumor:				0.594
Melanoma	46 (29)	27 (33)	19 (26)	
Non-small cell lung cancer	69 (44)	32 (38)	37 (49)	
Renal cell carcinoma	37 (23)	20 (25)	17 (22)	
Colorectal cancer	6 (4)	3 (4)	3 (4)	
ECOG-PS previous to SRT treatment				0.094
0–1	139 (98)	72 (88)	67 (97)	
2–3	13 (2)	11 (12)	2 (3)	
Present metastases				0.831
Oligometastatic (≤5 lesions)	64 (45)	33 (44)	30 (40.5)	
Polymetastatic (>5 lesions)	84 (55)	42 (56)	41 (55)	
Involved organs				0.423
1	38 (25)	24 (30)	14 (18)	
>1 (2–6)	116 (75)	57 (70)	73 (82)	
Targeted therapy				0.225
EGFRi	59 (37)	27 (33)	32 (43)	
mTKI	29 (18)	13 (16)	15 (20)	
VEGF(R)i	7 (4.4)	6 (7.4)	1 (1.4)	
mTORi	4 (2.5)	2 (2.5)	2 (2.7)	
ALKi	16 (10)	6 (7.4)	8 (11)	
BRAF(±MEK)i	43 (27)	27 (33)	16 (21.6)	
Start of targeted therapy				0.681
Before SRT	118 (77)	73 (90)	45 (61)	
time (days)	195 (5–1490)	200 (1–1000)	198 (7–1490)	
During/after SRT	37 (23)	8 (10)	29 (39)	
time (days)	7 (0–49)	-	7 (0–49)	
Treatment site, total	454	190	264	0.241
Cranial	374	160	214	
Extracranial:	80	30	50	
Lymph nodes	2	1	1	
Lung	27	12	15	
Abdomen	13	3	10	
Bone	34	12	22	
Soft tissue/muscle	4	2	2	
Prescribed dose (BED_10_, Gy)				
Brain SRT	63 (44–114)	63 (46.9–102)	62.1 (44–114)	0.216
SBRT	93 (53–180)	101.2 (54–180)	91.5 (53–159)	0.275
Tumor volume (mL)				
Cranial metastases	1 (0.03–23.9)	1.3 (0.04–15.3)	0.91 (0.03–23.9)	0.26
Extracranial metastases	7.5 (0.54–154.5)	11.6 (0.64–154.5)	6.4 (0.54–140.7)	0.437

**Table 2:** Correlation of clinical variables to the presence of severe (CTCAE v4.03 ≥ G3) toxicity present. Acute toxicity is ≤3 months after stereotactic radiotherapy (SRT), late toxicity is >3 months after SRT.

Correlation of Variables to Toxicity *	Acute Severe (≥G3) Toxicity			Late Severe (≥G3) Toxicity		
	No	Yes	*p*-Value	No	Yes	*p*-Value
Histology			0.133			0.829
Melanoma	62	1		60	3	
Non-small cell lung cancer	88	1		86	3	
Renal cell carcinoma	47	2		48	1	
Colorectal cancer	6	1		7	0	
ECOG-PS			0.408			0.387
0–1	185	4		182	7	
2–3	19	1		20	0	
Number of organs with metastatic disease at time of SRT						
0–3	143	5	0.16	144	4	0.366
>3	57	0		54	3	
Targeted therapy			0.074			0.107
EGFRi	66	2		65	3	
mTKI	32	1		33	0	
VEGF(R)i	14	0		14	0	
mTORi	3	1		3	1	
ALKi	28	0		28	0	
BRAF(±MEK)i	60	1		58	3	
Start of targeted therapy			0.905			0.646
Before SRT	158	4		157	5	
During/after SRT	46	1		45	2	
Targeted therapy paused during SRT			0.841			0.188
Yes	89	1 (2)		90	0 (0)	
No	114	4 (3)		111	7 (5)	
Prescribed dose (BED_10_, Gy)						
Brain SRT	65.5 (12)	61.9 (15)	0.56	65.2 (12)	70.3 (11)	0.37
SBRT	98.8 (33)	91.5 (0)	0.826	98.2 (33)	110.7 (27)	0.602
Tumor volume (cc)						
Cranial metastases	3.0 (5)	0.9 (0.4)	0.377	3.0 (5)	3.2 (4)	0.93
Extracranial metastases	19.5 (32)	11.1 (0)	0.794	19.0 (32)	27.6 (24)	0.706
Nr. of fractions			0.486			0.732
1	130	4		129	5	
>1	70	1		69	2	

* occurence within 208 stereotactic treatment sessions.

## Data Availability

The data presented in this study are available on request from the corresponding author.

## Supplementary Materials

The following are available online at https://www.mdpi.com/article/10.3390/cancers13194780/s1, Table S1: Specific reported toxicity that is probably or likely caused by the stereotactic radiotherapy (SRT) in combination with targeted therapy (TT) according to the CTCAE v4.03 scoring system. Table S2: Overview of examined targeted therapies within this study.
